# Potential of Essential Oils from *Cymbopogon winterianus* Jowitt: Promising Evaluation for the Control of Mollusks and Embryos of *Biomphalaria glabrata* and *Schistosoma mansoni* Cercariae

**DOI:** 10.3390/ph18030318

**Published:** 2025-02-25

**Authors:** Keyla Nunes Farias Gomes, Francisco Paiva Machado, Ester Maria Mota, Ana Cláudia Rodrigues da Silva, Mikaella Gonçalves Xavier, Joana Tostes da Cunha e Menezes, Anita Ferreira do Valle, Leandro Louback da Silva, Beatriz de Frias Leite, Leandro Rocha, Robson Xavier Faria

**Affiliations:** 1 Postgraduate Program in Plant Biotechnology and Bioprocesses, Center of Health Sciences, Federal University of Rio de Janeiro, Carlos Chagas Filho Avenue 373, University City, Rio de Janeiro 21941-902, RJ, Brazil; keylafariasgomes@hotmail.com (K.N.F.G.); lean.machado@gmail.com (L.R.); 2 Natural Products Technology Laboratory, Federal Fluminense University, Mário Viana Street 523, Niteroi 24241-002, RJ, Brazil; fmachado@id.uff.br; 3 Environmental Health Assessment and Promotion Laboratory, Oswaldo Cruz Institute, Brazil Avenue 4365, Rio de Janeiro 21040-900, RJ, Brazil; 4 Postgraduate Program in Sciences Applied to Natural Health Products, Fluminense Federal University, Rua Mario Viana, 523, Niteroi 24241-000, RJ, Brazil; 5 Laboratory of Experimental Medicine and Health, Oswaldo Cruz Foundation (FIOCRUZ), Brazil Avenue 4365, Rio de Janeiro 21040-900, RJ, Brazil; mota.ester@gmail.com; 6 Laboratory of Applied Studies in Photosynthesis, Department of Biochemistry, Federal University of Rio de Janeiro, Rio de Janeiro 21941-909, RJ, Brazil; anacrs1@yahoo.com.br (A.C.R.d.S.); mikaellagoncalves25@gmail.com (M.G.X.); tostesjoana55@gmail.com (J.T.d.C.e.M.); avalle@iq.ufrj.br (A.F.d.V.); 7 Laboratory of Pharmacology of Bioactive Products, Institute of Pharmaceutical Sciences, Federal University of Rio de Janeiro, Macaé 27930-560, RJ, Brazil; lelouback@hotmail.com (L.L.d.S.); beatrizfriasufrj@gmail.com (B.d.F.L.)

**Keywords:** schistosomiasis, alternative control, natural product, intermediate host

## Abstract

**Background/objectives:** Schistosomiasis is a parasitic disease that represents a serious public health problem. An alternative for the control of snails, intermediate hosts of schistosomiasis, is the use of molluskicides. Niclosamide, recommended by the WHO, has limitations, such as environmental toxicity, which has driven the search for safer and biodegradable alternatives, especially of plant origin. In this context, this study investigated the biological activity of *Cymbopogon winterianus* essential oil on embryos, juveniles, and adults of *Biomphalaria glabrata* and cercariae of *Schistosoma mansoni*. **Methods:** Essential oils (EOs) were extracted from fresh leaves via the Clevenger system and characterized via gas chromatography (GC/MS and GC/FID), revealing geraniol (25.0%), citronellal (29.2%), citronellol (10.5%) and elemol (9.6%) as the main components. **Results:** The results revealed lethal concentrations 90 (LC_90_) for young and adult snails of 60.72 mg/L, 74.21 mg/L and 115.35 mg/L, respectively. In the histological analysis, no changes were observed in the tissues of the mollusks exposed to the lethal concentration 25 (LC_25_). However, the lethal concentrations 50 (LC_50_) and 75 (LC_75_) caused crystalline concretions in proximity to the renal saccular portion. At a concentration of 60 mg/L, the oil resulted in 100% lethality in embryos and cercaricidal activity greater than 90% in 3 h. Acute toxicity tests in mice via the intraperitoneal or oral route did not reveal toxic effects, with hematological and biochemical parameters within the reference values. Furthermore, the oil did not inhibit acetylcholinesterase (AChE), indicating low toxicity to fish, and caused a slight reduction in human butyrylcholinesterase (hBChE) activity without affecting human AChE, which suggests low toxicity to mammalian tissues. In terms of environmental impact, the oil was not toxic to algae until the 75th day, with mortality observed thereafter. **Conclusions:** These results indicate that essential oils have great potential as biodegradable and safe alternatives for controlling mollusks and interrupting the schistosomiasis cycle.

## 1. Introduction

Schistosomiasis is a disease that represents a serious public health problem. This zoonotic infection is caused by trematode parasites of the genus Schistosoma and affects the most vulnerable populations, impacting human health and socioeconomic development. This disease, which is considered the second most significant parasitic infection after malaria, is one of the main neglected tropical diseases (NTDs), a group of infectious diseases that prevail in tropical and subtropical regions and mainly affect poor populations with limited access to health care, sanitation, and resources for prevention and treatment [1]. Its consequences for health and socioeconomic progress are particularly severe in tropical countries, and effective strategies for its control are needed [2,3].

Schistosomiasis, also known as snail disease, bilharzia, schistosomiasis, or water belly, is a widely disseminated parasitic infection. It is present in 78 countries, 52 of which are experiencing moderate to high levels of transmission, making it one of the most prevalent parasitic diseases worldwide. According to the World Health Organization [4], approximately 240 million people are affected by schistosomiasis worldwide, and more than 700 million live in endemic areas.

The disease is endemic in North and South America and areas of Africa and Asia [5]. In Brazil, the most affected country in Latin America, approximately 1.5 million people are infected, with the highest concentration of cases in Northeast China [6].

This disease is directly related to the presence of snails that serve as intermediate hosts. Thus, controlling these snails is crucial for preventing disease [7]. In Brazil, infection is currently caused by the species *S. mansoni*, and three species of snails play the role of intermediate hosts: *Biomphalaria tenagophila*, *Biomphalaria straminea* and *Biomphalaria glabrata*. Among them, *B. glabrata* is the most widely distributed and the most susceptible to infection by the parasite [7,8].

An alternative for controlling these snails, which are intermediate hosts of schistosomiasis, is the use of synthetic molluskicides. Niclosamide is the substance recommended by the WHO for this purpose. This synthetic substance acts mainly by inhibiting oxidative phosphorylation in the parasite. Niclosamide has low solubility in water, and the crystalline form of the drug is not absorbed in effective therapeutic amounts; therefore, it exerts its effects mainly on the gastrointestinal tract. However, the use of niclosamide presents challenges, such as high cost, toxicity to non-target organisms, and low selectivity, which limit its practical application [8,9,10].

In this context, the scientific community and industry have shown growing interest in the development of biodegradable products, with a focus on plant-based alternatives. Ideally, these products should be selective for snails of the genus Biomphalaria, minimizing toxicity to the environment and non-target species. A promising approach is the research and development of new plant-derived products [7,11,12].

As an example of a prototype molluskicide of natural origin, the latex of *E. milii* resulted in the development of MoluSchall, a prototype kit that has proven effective in controlling mollusks. The product showed good stability for 24 months and low toxicity to *Danio rerio*, being lethal to three species of mollusks and effective against *B. glabrata* under seminatural conditions, with a performance similar to that observed in the laboratory. Thus, MoluSchall is a natural, effective and low-cost molluskicide with the potential to control the transmission of *S. mansoni* with less environmental impact [13].

Therefore, in this work, we investigated the molluskicidal activity of the species *Cymbopogon winterianus*. These EOs have gained prominence for diverse applications, including pesticides. This species is popularly known as citronella or citronella grass and belongs to the Poaceae family [14,15,16]. This plant is commonly found in tropical and subtropical countries in places close to mountains, plains and arid areas. Citronella is resistant to various climatic conditions, making it easy to grow [17,18,19]. Studies have demonstrated that the leaves of the species *C. winterianus* have anti-inflammatory [20], antibacterial [16], antifungal [21], anticonvulsant and, mainly, larvicidal activities against *Aedes aegypti* [22,23].

The objective of the present work was to investigate the biological activity of embryos and young and adult stages of the species *Biomphalaria glabrata* and cercariae of *Schistosoma mansoni*.

## 2. Results

### 2.1. EO Characterization

**Figure 1:**
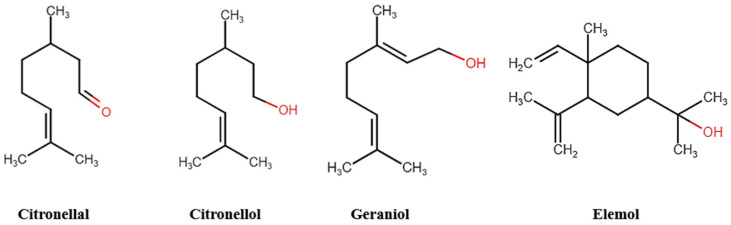
Major compounds of *Cymbopogon winterianus* EO.

The extracted EO yield was 1.27% (*w*/*w*). The chemical analysis of *Cymbopogon winterianus* EO in Table 1 shows that 92.9% of the compounds were present in the oil. Among the main constituents, citronellol (29.2%), geraniol (25.0%), citronellol (10.5%), and elemol (9.6%) stand out, as illustrated in Figure 1.

### 2.2. Molluscicidal Assay

**Figure 2:**
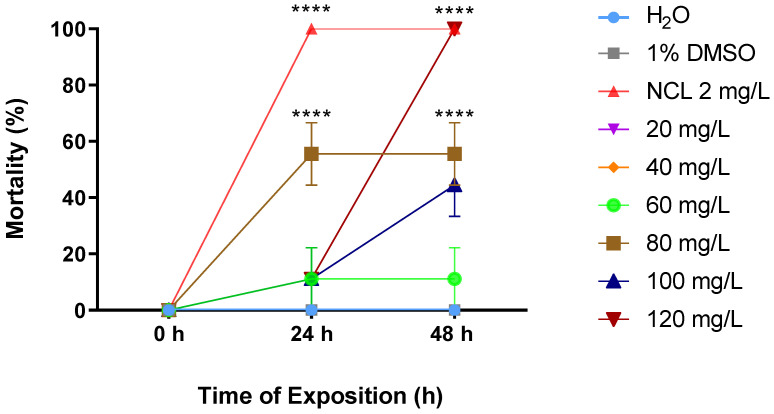
Mortality rate of *Cymbopogon winterianus* EO on the adult snail *Biomphalaria glabrata* (10–12 mm). This experiment was performed in triplicate on at least 3 different days. The results expressed in the graph represent the mean ± standard error. **** *p* < 0.0001.

**Figure 3:**
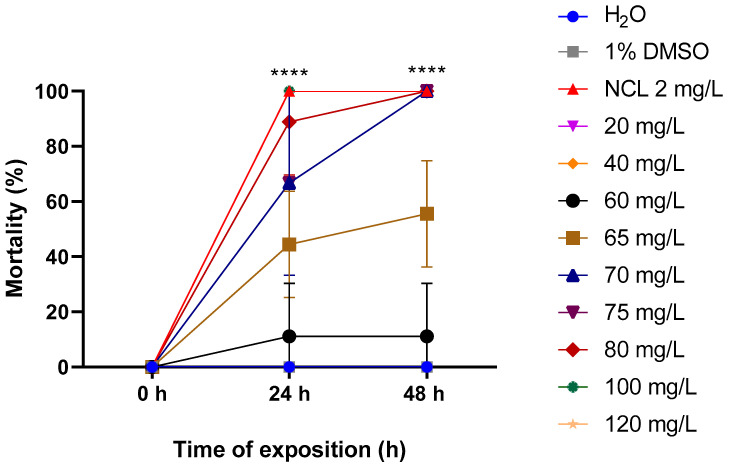
Mortality rate of *Cymbopogon winterianus* EO on the young snail *Biomphalaria glabrata* (6–8 mm). This experiment was performed in triplicate on at least 3 different days. The results expressed in the graph represent the mean ± standard error. **** *p* < 0.0001.

**Figure 4:**
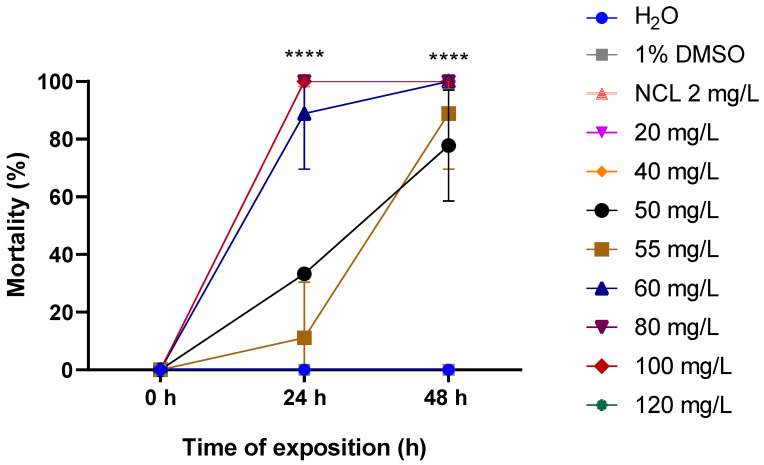
Mortality rate of *Cymbopogon winterianus* EO on the young snail *Biomphalaria glabrata* (3–5 mm). This experiment was performed in triplicate on at least 3 different days. The results expressed in the graph represent the mean ± standard error. **** *p* < 0.0001.

The EO extracted from the leaves of *Cymbopogon winterianus* was lethal to snails of the species *Biomphalaria glabrata* both in the young and adult stages. At concentrations of 80 mg/L and 120 mg/L, the lethality rates were 55.55% and 100%, respectively, after 48 h for adult snails measuring 10–12 mm (Figure 2). For young snails measuring 6–8 mm, 100% mortality was observed at a concentration of 70 mg/L after 48 h (Figure 3). For smaller young snails measuring 3–5 mm, the mortality rate was 100% at a concentration of 60 mg/L during the same period (Figure 4). Furthermore, it was possible to calculate the lethal concentrations for 50% (LC_50_) and 90% (LC_90_) deaths in both stages of development, as shown in Table 2.

### 2.3. Biochemical Analysis

Analysis of the biochemical parameters revealed that the glucose levels were significantly greater in the treatments than in the negative control (water). Specifically, at the lethal concentration of 75 (LC_75_), the glucose levels in the treated groups were comparable to those observed in the positive control, suggesting a potential metabolic response to the experimental conditions or the substances used. This increase in glucose levels may indicate a physiological stress response or alterations in energy metabolism associated with the treatments.

**Figure 5:**
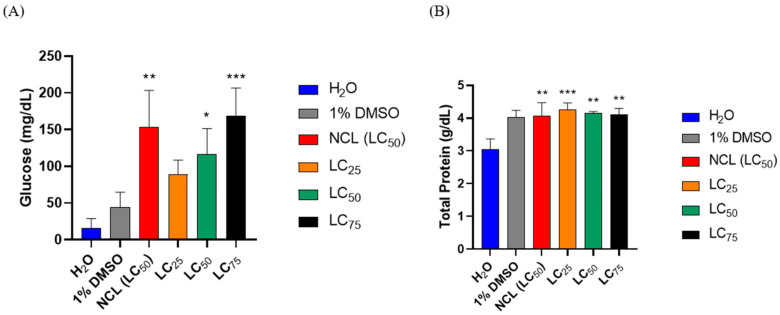
Analysis of the hemolymph of *Biomphalaria glabrata* snails 48 h after exposure to EOs from the species *Cymbopogon winterianus* and the corresponding negative control groups (water (H_2_O) and dimethyl sulfoxide (DMSO)) and positive control groups (niclosamide (NCL)). (**A**) Glucose; (**B**) total protein. The lethal concentration 50 (LC_50_) of NCL was 0.06 mg/L; the lethal concentration 25 (LC_25_) was 62.21 mg/L; the lethal concentration 50 (LC_50_) was 82.65 mg/L; and the lethal concentration 75 (LC_75_) was 103.09 mg/L. The results expressed in the graph represent the mean ± standard error. * *p* = 0.0157; ** *p* = 0.0019; *** *p* = 0.009.

In addition, total protein analysis revealed that the treatments involving EO resulted in protein concentrations similar to one another, with no significant differences across these groups. However, a clear distinction was observed compared with the negative control (water), which presented lower protein levels. These findings suggest that the presence of EO may influence protein synthesis or stability, potentially reflecting an adaptive response to the treatment. The results are illustrated in Figure 5, where the biochemical data for glucose and total protein levels are presented for further comparison.

### 2.4. Histopathological Analysis of Species Biomphalaria glabrata

**Figure 6:**
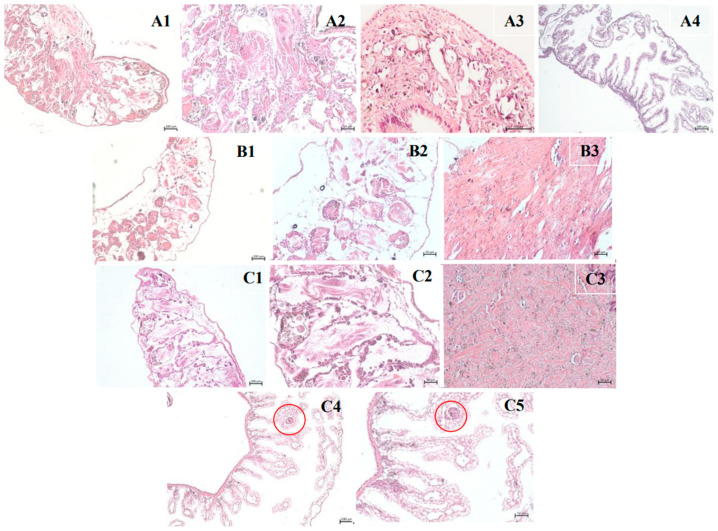
(**A1**–**A4**)—*Biomphalaria glabrata* negative control group (H_2_O) ovotestis region (100 µm and 50 µm), muffle (50 µm) and kidney (100 µm) without alterations, respectively; (**B1**–**B3**)—negative control group (1% DMSO) ovotestis region (100 µm and 50 µm) and muffle (50 µm) without alterations, respectively; (**C1**–**C5**): positive control group (NCL) ovotestis region (100 µm and 50 µm), muffle (50 µm) and kidney (100 µm and 50 µm) with tissue alterations, respectively.

**Figure 7:**
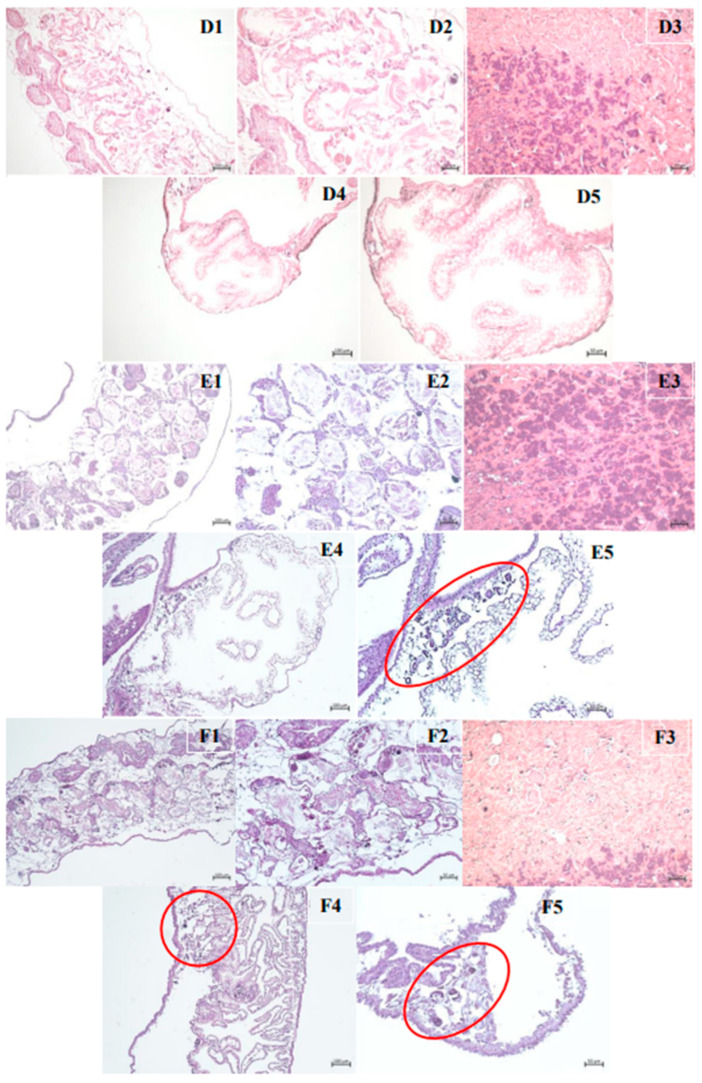
(**D1**–**D5**)—*Biomphalaria glabrata* treatment group (CL_25_) ovotestis region (100 µm and 50 µm), muffle (50 µm) and kidney (100 µm and 50 µm); (**E1**–**E5**)—treatment group (CL_50_) ovotestis region (100 µm and 50 µm), muffle (50 µm) and kidney (100 µm and 50 µm); (**F1**–**F5**): treatment group (CL_75_) ovotestis region (100 µm and 50 µm), muffle (50 µm) and kidney (100 µm and 50 µm).

The results of the histological analyses are shown in Figure 6 and Figure 7. Images 6A1, 6A2, 6A3, and 6A4 show the ovotestis, muffle, and kidney regions of *B. glabrata* in the control group, which was treated with water. Images 6B1, 6B2 and 6B3 represent the ovotestis and muffle regions of the control group treated with 1% DMSO. Both control groups showed no changes. However, Figure 6 (C1–C5) show longitudinal sections of the ovotestis, muffle, and kidney of the positive control group, treated with niclosamide, where small clusters of cells are observed in the renal tubules (marked in red).

In the tissues of mollusks treated with the lethal subconcentration 25 (LC_25_) of *C. winterianus* EO, images D1, D2, D3, D4, and D5 (ovotest, muffle and kidney) did not show any histological changes. With respect to the tissues treated with the lethal subconcentration 50 (LC_50_), in images E1, E2 (ovotest), and E3 (muffle), it was not possible to observe tissue changes. However, in images E4 and E5 (kidney), crystalline concretions (marked in red) were observed close to the renal saccular portion. Similar changes were also highlighted in images F4 and F5 (kidney), which were obtained after treatment with the lethal subconcentration 75 (LC_75_).

### 2.5. Toxicity Assay on Species Biomphalaria tenagophila and Physella acuta

**Figure 8:**
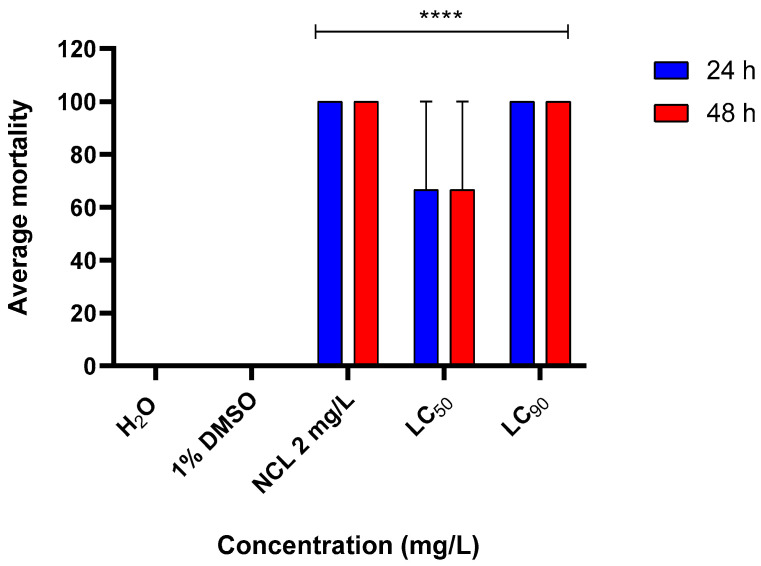
Mortality rate of *Biomphalaria tenagophila* mollusks affected by *Cymbopogon winterianus* EO (N = 3). This experiment was performed in triplicate on at least 3 different days. The lethal concentration 50 (LC_50_) corresponds to 82.65 mg/L; the lethal concentration 90 (LC_90_) corresponds to 115.35 mg/L. The results expressed in the graph represent the mean ± standard error. **** *p* < 0.0001.

**Figure 9:**
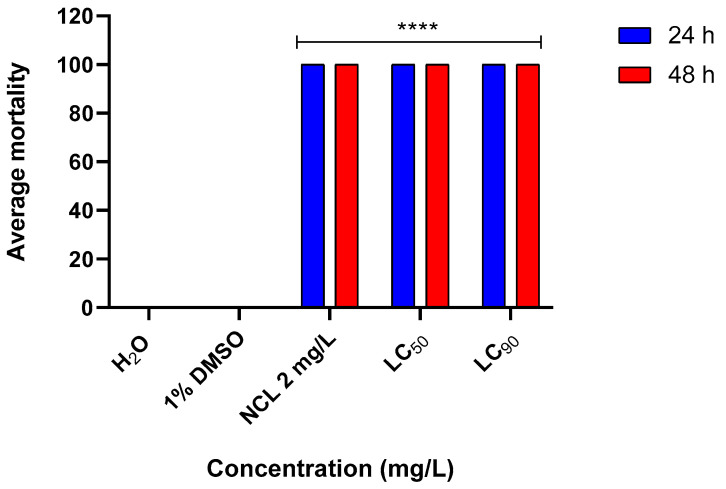
Mortality rate of *Physella acuta* mollusks caused by *Cymbopogon winterianus* EO (N = 3). This experiment was performed in triplicate on at least 3 different days. The lethal concentration 50 (LC_50_) corresponds to 82.65 mg/L; the lethal concentration 90 (LC_90_) corresponds to 115.35 mg/L. The results expressed in the graph represent the mean ± standard error. **** *p* < 0.0001.

In the environmental toxicity test, we observed that the EO resulted in 100% mortality in *Biomphalaria tenagophila* at a lethal concentration of 90% (LC_90_) (Figure 8). For the species *Physella acuta*, the lethality was 100% at lethal concentrations of 50 and 90 for 24 h (Figure 9).

### 2.6. Ecotoxicity in Algae

According to Table 3 and Table 4, the EO of *C. winterianus* did not have toxic effects on algae until the thirtieth day of observation. However, from the seventy-fifth day onward, the algae (mollusk reservatory) died after exposure to the EO.

### 2.7. Ovicidal Assay

**Figure 10:**
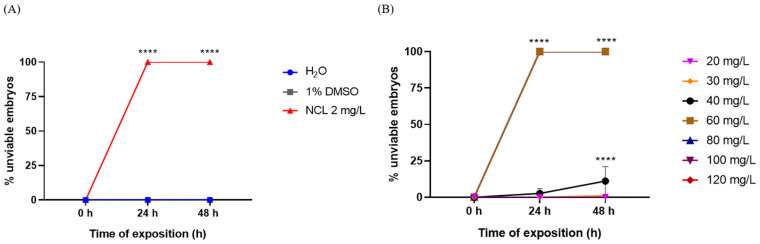
Relationship between the percentage of unviable embryos in the negative control water or 1% dimethyl sulfoxide or the positive control niclosamide after 24 h and 48 h of the experiment. Relationships between the percentage of unviable *Cymbopogon winterianus* embryos and the number of EO-treated *Cymbopogon winterianus* embryos after 24 h and 48 h of treatment. (**A**) Control; (**B**) *Cymbopogon winterianus* EO treatment. This experiment was carried out in triplicate on at least 3 different days. The results expressed in the graph represent the mean ± standard error. **** *p* < 0.0001.

In the ovicidal assay, the EO of *Cymbopogon winterianus*, at a concentration of 60 mg/L, induced 100% lethality in embryos of the species *Biomphalaria glabrata* after 48 h. This treatment significantly differed from the negative control (H_2_O). The 50% and 90% lethal concentrations of EO were 51.21 mg/L and 81.30 mg/L, respectively (Figure 10).

### 2.8. Cercaricidal Assay

**Figure 11:**
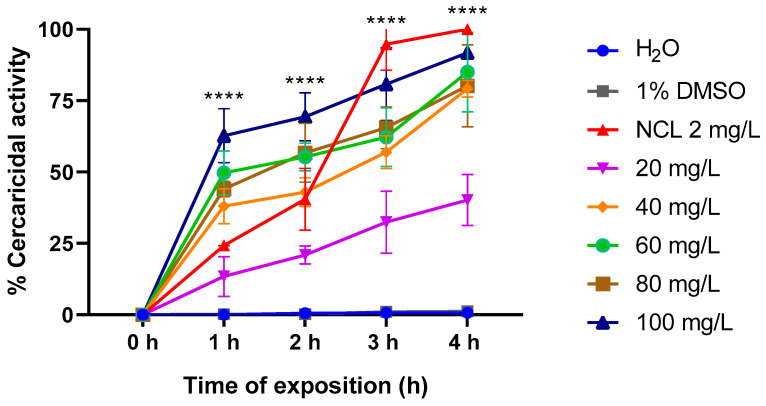
Relationships between the cercaricidal activity rates of the negative controls (water and 1% dimethyl sulfoxide) and the positive control (niclosamide) after 0 h, 1 h, 2 h, 3 h, and 4 h of the experiment. Relationships between cercaricidal activity and EO of *Cymbopogon winterianus* after 0 h, 1 h, 2 h, 3 h and 4 h of treatment. This experiment was performed in triplicate on at least 3 different days. The results expressed in the graph represent the mean ± standard error. **** *p* < 0.0001.

In the cercaricidal activity test, the EO demonstrated the following results: 62.72% efficacy after 1 h, 69.39% efficacy after 2 h, 80.81% efficacy after 3 h, and 91.81% efficacy after 4 h at a concentration of 100 mg/L. In comparison, the positive control, niclosamide, presented a lower activity than the EO during the first two hours of the experiment. The last hour of the experiment (4 h) presented 100% efficacy. We calculated 50 and 90 lethal concentrations of the EO, with values of 34.51 mg/L and 82.67 mg/L, respectively (Figure 11).

### 2.9. In Vivo Acute Toxicity Assay

In the acute toxicity test, after 24 h, the EO showed no signs of toxicity either orally or intraperitoneally (Table 5).

### 2.10. Hematological and Biochemical Analysis

Hematological and biochemical analyses of Swiss Webster mice treated with 0.9% saline solution (100 μL/animal), dimethyl sulfoxide (DMSO), or *Cymbopogon winterianus* EO were compared with those of the saline control group and the reference values (Table 6).

The red blood cell counts and hemoglobin levels in the group treated with the EO oil were slightly lower than those observed in the saline control groups but still within the reference values. The hematocrit evaluation also revealed lower values in the group treated with the EO than in the control group but was still within the normal range. The mean corpuscular volume (MCV) and mean corpuscular hemoglobin (MCH) indices were consistent across all groups and remained within the reference values. The mean corpuscular hemoglobin concentration (MCHC) was also within the reference values for both the treatment and control groups.

The leukocyte count in the EO-treated group was slightly greater than that in the saline and DMSO control groups. However, the leukocyte counts in all the groups remained within the reference values. The platelet count in the EO-treated group was greater than that in the saline and DMSO control groups. However, both the treated and control groups were within the reference values.

For the biochemical parameters, the albumin levels were high compared with those of the controls, but all the levels remained within the reference values. For the calcium levels, both the EO and the dimethyl sulfoxide had similar values, whereas the saline control had a slightly greater value. The saline control and EO groups presented increased levels of aspartate aminotransferase (AST) and alanine aminotransferase (ALT), suggesting increased hepatic stress. In contrast, the aspartate aminotransferase (AST) and alanine aminotransferase (ALT) levels in the group treated with dimethyl sulfoxide were within the reference range. Lactate dehydrogenase (LDH) levels varied between the groups, with the essential oil showing the highest levels. Both alkaline phosphatase and creatinine levels were within the reference values.

### 2.11. Effect of Cymbopogon winterianus EO on Acetylcholinesterase

EO treatment did not inhibit the eeAChE or hAChE enzymes, which maintain their full activity in water (H_2_O). Administration of the inhibitor rivastigmine almost completely reversed the increase in enzyme activity. Furthermore, the diluent DMSO had no effect on enzyme function. Therefore, it is likely that this essential oil is not toxic to fish or that it acts via a mechanism independent of AChE.

In terms of human enzymes, the essential oil slightly reduced hBChE activity and did not act on hAChE. Therefore, these data reinforce the low toxicity of the essential oil for mammalian tissues (Table 7).

## 3. Discussion

In our study, we extracted the EO of *Cymbopogon winterianus* via hydrodistillation via a Clevenger-type apparatus and obtained a yield of 1.27%. Rodrigues and collaborators (2013) [24], who used the same methodology, obtained a yield of 1.3%, confirming our results.

The main metabolites found in the EO were citronellal (29.2%), geraniol (25.0%), citronellol (10.5%), and elemol (9.6%). Therefore, these results corroborate other studies that described these compounds as major constituents, although in different proportions [24,25,26].

The differences observed in the composition and concentration of EOs from the same species can be attributed to variations in chemotypes and biotic interactions, such as those between microorganisms and insects. Environmental and cultivation factors, including collection time and date, age, and stage of the plant, also influence plant composition. Genetic, environmental, and ecophysiological parameters can cause these variations. Understanding these influences is crucial for optimizing the use and application of EOs [24,25].

The molluskicidal properties of the genus *Cymbopogon* have been extensively investigated. According to Pereira and collaborators (2020) [27], the efficacy of several species of this genus (*Cymbopogon citratus* DC. Stapf., *Cymbopogon nervatus* (Hochst.) Chiov. and *Cymbopogon winterianus* Jowitt) was evaluated against mollusks of the species *Bulinus truncatus*, *Biomphalaria pfeifferi*, *Biomphalaria tenagophila,* and *Biomphalaria glabrata.*

Rodrigues et al. (2013) [24] investigated the activity of EOs on mollusks of the species *B. glabrata* following the methodology recommended by the WHO (1983) [28]. Their study used adult mollusks with a diameter between 10 and 15 mm. In contrast, the present research followed the methodology of Santos et al. (2017) [29], in which we used 24-well plates to evaluate molluskicidal activity. In our study, we worked with mollusks at different stages of development, being both young (3–5 mm and 6–8 mm) and adult (10–12 mm) individuals. This allowed us to use smaller mollusks and EOs during the evaluation.

In the study by Rodrigues (2013) [24], the lethal concentration values for the EO were as follows: LC_90_ = 569.4 mg/L at 24 h, LC_50_ = 125.8 mg/L at 48 h, and LC_20_ = 97.4 mg/L at 72 h. In the present research, we evaluated the lethal concentration 90 for different stages of mollusk development for 48 h. Our results revealed that the lethal concentration of 90 for adult mollusks (10–12 mm) was 135.35 mg/L, whereas for young mollusks, the lethal concentration of 90 was significantly lower: 74.21 mg/L for 6–8 mm mollusks and 60.78 mg/L for 3–5 mm mollusks.

In a histological analysis, a study with the EO *C. winterianus* revealed the formation of crystalline concretions in mollusks treated with subconcentrations (LC_50_ and LC_75_), a phenomenon similar to that described by the PAM (1958) [30]. These findings may indicate a physiological or toxic response of mollusks, possibly associated with metabolic or excretory processes. In comparison, a study by Alberto-Silva (2022) [31] on *Biomphalaria glabrata* infected by *Angiostrongylus cantonensis* and exposed to latex from *Euphorbia milii* var. hislopii revealed alterations in the gonadal system and reduced fecundity. Although both studies reported adverse effects caused by treatments originating from natural products, the mechanisms and types of damage observed are distinct; the first involves the formation of crystals, whereas the second affects the reproductive system of mollusks. Both agents analyzed cause significant damage but in different physiological contexts.

Few studies have highlighted the toxicity of substances in the *B. glabrata* tissue; thus, we mentioned the main regions with structural disruptions. The muffle toxicity may be associated with local contact between EO and cephalopodal region after oral entry. The kidney toxicity may be related to the mollusk’s tentative excretion of the EO. Ovotestis toxicity can be caused by EO accumulation in the fat present in this region. The alterations observed in the kidneys and reproductive tissues were compatible with previously described for Pile et al. (2002) [32]. Although the literature exhibits histological disruption mainly in the reproductive system, these publications are associated with *B. glabrata* infection. Therefore, our experimental model distinguishes between tropism for reproductive tissues.

Additionally, we performed assays with *Biomphalaria glabrata* embryos and *Schistosoma mansoni* cercariae. Our results demonstrated that *Cymbopogon winterianus* EO has effective activity, resulting in 100% lethality for embryos at a concentration of 60 mg/L and approximately 90% lethality for *S. mansoni* cercariae. In contrast, Martins et al. (2022) [33] investigated the effects of 3-aryl-2-hydroxy-1,4-naphthoquinones (NAs) on *B. glabrata* and *S. mansoni* and reported that these naphthoquinones had slight effects on both embryos and cercariae. These results highlight that not all substances are effective at all cycle stages, highlighting the need for a more detailed approach, considering the variability in activity between different stages of the schistosomiasis cycle.

Costa et al. (2015) [25] studied the EO of *Cymbopogon winterianus*. They observed molluskicidal activity against *Biomphalaria tenagophila* and *Lymnaea columella*, with minimum effective concentrations of 20 ppm and 30 ppm, respectively. In our study, we tested the same EO on *Physella acuta* and *Biomphalaria tenagophila* and observed 100% lethality at a lethal concentration of 90 (115.35 mg/L) for both species.

Compared with the results of Costa et al. (2015) [25], our data indicate a lethal concentration higher than that for *L. columella* but similar to or even lower than that for *B. tenagophila*. This may reflect differences in species sensitivity or methodology. Our findings suggest that *Cymbopogon winterianus* EO effectively controls mollusks, offering a potentially environmentally friendly alternative to traditional chemical methods.

Assessing the toxicity of EOs to algae is essential for understanding their long-term environmental impact, as algae are important bioindicators of water quality [34]. Although the essential oil did not cause toxicity until day 30, mortality in algae was observed from day 75 onward. These results highlight the need to monitor long-term effects to ensure that EOs do not cause significant negative impacts on aquatic ecosystems, especially in environments where algae interact with mollusks and other organisms.

Changes in metabolic glucose and total protein levels were observed in mollusks exposed to lethal subconcentrations of *Cymbopogon winterianus* EO. In our study, an increase in glucose levels was observed at subconcentrations of 50 and 75 mg/L, as well as in the positive control, niclosamide. This increase in glucose consumption was previously reported by Mello-Silva (2006, 2010) [35,36] in studies on the hemolymph of infected mollusks exposed to *Euphorbia splendens* latex. The change in glucose levels is likely a result of the increased energy demand induced by exposure to the oil, which may lead to an increase in ATP consumption and an acceleration of glycolysis.

For the total protein content, an increase in the lethal subconcentrations of the EO was observed compared with that of the negative control (water). This increase is consistent with the results of Mello-Silva et al. (2006) [35], who reported an increase in total protein in mollusks exposed to *Euphorbia splendens* latex. This increase may be associated with the acceleration of gluconeogenesis, a response to the progressive level of intoxication caused by the higher concentrations of the EO tested.

The tested EO did not inhibit the enzymes eeAChE (fish acetylcholinesterase) and hAChE (human acetylcholinesterase), suggesting that it is either nontoxic to fish or acts via a mechanism other than AChE inhibition. Acetylcholinesterase plays a vital role in regulating neurotransmission by degrading acetylcholine and preventing excessive stimulation of neurons [37]. Inhibition of this enzyme can lead to significant neurotoxic effects.

For human enzymes, the EO slightly reduced the activity of hBChE (human butyrylcholinesterase) without affecting hAChE. Although butyrylcholinesterase also degrades cholinergic molecules, its role is less central to neuromuscular transmission than acetylcholinesterase [38]. The observed slight reduction in hBChE activity, combined with the lack of effect on hAChE, reinforces the low toxicity of the EO to mammalian tissues.

These results are consistent with those of previous studies on EO. Hung et al. (2022) [38] reported variations in AChE inhibitory activity among different EOs, suggesting the importance of choosing oils with low toxicity potential. Johnson et al. (2021) [37] highlighted the potential of *Cymbopogon citratus* as a source of natural AChE inhibitors, reinforcing the need to evaluate its inhibitory activity carefully to avoid adverse effects.

Therefore, the lack of significant AChE inhibition and the slight reduction in hBChE activity caused by the EO suggest a low probability of neurotoxic effects. These findings indicate that essential oils are relatively safe for aquatic environments and mammalian tissues, although further studies are needed to confirm their safety in practical applications.

Additionally, we performed acute toxicity tests of *Cymbopogon winterianus* EO via the intraperitoneal and oral routes in mice. No signs of toxicity were observed in the biochemical or hematological tests after the administration of the EO. These results are important because they demonstrate that, despite the molluskicidal efficacy of the EO, it does not present significant adverse effects in mammals. The absence of toxicity in mice reinforces the potential safety of this EO for use in environments where human and animal health must be considered. Therefore, *Cymbopogon winterianus* EO appears to be a promising alternative for controlling disease-causing mollusks, offering an effective and safe solution compared with other chemical agents that may have undesirable side effects.

## 4. Materials and Methods

### 4.1. Collect

Leaves of *Cybompogon winterianus,* cultivated and collected by Dr. José Augusto Albuquerque dos Santos at Sítio do Gravatá, Unamar, Tamoios, 2nd District of Cabo Frio, Rio de Janeiro, Brazil (S 22°37′26′′ and W 42°01′19′′), on 14 March 2023, were registered under number AF3CD16 in the SisGen system by the Laboratory for the Assessment and Promotion of Environmental Health (LAPSA). Dr. Marcelo Neto Galvão identified them at the Center for Innovation in Biodiversity and Health within the Botanical Collection of Medicinal Plants (CBPM) at Farmanguinhos/Fiocruz, with registration number 1663. 

### 4.2. EO Extraction

Leaves of the species *C. winterianus* (228 g) were crushed in distilled water. After this step, the plant material was immediately placed in a 5 L round-bottomed flask and subjected to hydrodistillation for 3 h using a Clevenger apparatus. The EO obtained was subsequently dried over anhydrous sodium sulfate and stored in an amber glass bottle at 4 °C.

### 4.3. Chemic Characterization of EO from Leaves of Cymbopogon winterianus

The EO was chemically characterized via a GC-MS QP2010 gas chromatograph (Shimadzu) with a mass spectrometer and a GC-2014 gas chromatograph (Shimadzu) equipped with a flame ionization detector (FID). The conditions for gas chromatography (GC) were as follows: injector temperature, 260 °C; carrier gas, helium; flow rate, 1 mL/min; and split injection ratio, 1:40. The oven was initially heated at 60 °C and then heated to 290 °C at a rate of 3 °C/min. One microliter of the sample dissolved in hexane (1:100 mg/μL) was injected into an RTX-5 column (0.25 mm ID, 30 m length, 0.25 μm film thickness). Electron ionization mass spectrometry (MS) was performed at 70 eV, with a scan rate of one scan per second. The GC-FID conditions were similar to those of MS, except for the FID temperature, which was 290 °C. The arithmetic index (AI) was calculated by interpolating the retention times of a mixture of aliphatic hydrocarbons (C9–C30) analyzed under the same conditions. The substances were identified by comparing the retention indices and mass spectra with those described in the literature [39] and the MS fragmentation patterns with NIST mass spectral libraries. The relative abundances of the chemical constituents were determined via GC-FID, and the percentages of the compounds obtained via normalization to the FID peak areas were analyzed.

### 4.4. Biological Assays

#### 4.4.1. Raising and Maintenance of Mollusks

Mollusks of the species *Biomphalaria glabrata* were collected in Sumidouro, Rio de Janeiro, RJ, Brazil (S 22°02′59′′ and W 42°40′29′′). These mollusks were raised and maintained in 200-L tanks with dechlorinated water. The food consisted of fresh lettuce leaves provided three times a week at the Lauro Travassos Pavilion, which is located on the Oswaldo Cruz Foundation Campus (coordinates S 22°52′33′′ and W 43°14′46′′). To ensure an adequate quantity of mollusks for laboratory needs, Styrofoam plates with approximate dimensions of 10 cm × 10 cm were added to the tanks to obtain embryos.

#### 4.4.2. Molluscicidal Assay

The experiments were conducted in 24-well plates following the methodology described by Santos et al. (2017) [29]. Adult snails of the species *Biomphalaria glabrata* with shell diameters of 10–12 mm and young snails with diameters of 6–8 mm and 3–5 mm, all not infected by *Schistosoma mansoni*, were used. Each snail was placed individually in a well of the plate. The snails were exposed to negative controls (distilled water and 1% DMSO), positive controls (Niclosamida^®^ (NCL) at 2 mg/L), and concentrations of 10, 20, 40, 60, 80, and 100 mg/L EO in a final volume of 2 mL (2000 µL) per well. The snails were exposed to the treatment for 24 h and 48 h. The experiments were performed in triplicate and on at least three different days. Mollusk mortality was identified by observing the condition of the shells, lack of mobility, odors, retraction of the soft part, absence of heartbeats, and release of hemolymph [40].

### 4.5. Biochemical Analysis

After treatment with subconcentrations (LC_25_, LC_50_, and LC_75_) of the tested agents, the hemolymph of five snails per group was collected via cardiac puncture and stored in Eppendorf microtubes. The samples were analyzed on the same day. The parameters evaluated were glucose and total protein. The methodology used is described in the Analisa laboratory diagnostic kit.

### 4.6. Histopathological Analysis of Species Biomphalaria glabrata

Following treatment at subconcentrations (LC_25_, LC_50_, and LC_75_), the shells of five snails were removed, and their soft tissues were preserved in a 10% formalin solution (Carson’s Millonig) for 24 h. Afterward, the tissues were dehydrated in increasing concentrations of ethanol (30%, 50%, 70%, and 90%) and clarified with xylene. The samples were then embedded in liquid paraffin at 60 °C. Serial longitudinal sections, each 5 µm thick, were prepared via a microtome. The sections were stained with hematoxylin-eosin (HE) and examined under a Zeiss Axio Scope microscope.

### 4.7. Toxicity Assay on Species Biomphalaria tenagophila and Physella acuta

#### 4.7.1. Raising and Maintenance of Mollusks

Adult mollusks of the species *Biomphalaria tenagophila* (Orbigny, 1835) and *Physella acuta* (Draparnaud, 1805) were collected from drainage channels at the Oswaldo Cruz Foundation—Fiocruz, Manguinhos campus, Rio de Janeiro, RJ (coordinates S 22°52′45′′ and W 43°14′45′′). The plants were kept in plastic containers with dechlorinated water and fed lettuce leaves three times a week. The mollusks were kept in these containers for 48 h before being used in the bioassay. The mollusk collection was approved on November 21, 2024, by the Biodiversity Authorization and Information System with registration number 86286-1.

#### 4.7.2. Molluscicidal Assay of Species *Biomphalaria tenagophila* and *Physella acuta*

Adult snails of *Biomphalaria tenagophila* and *Physella acuta* were exposed to lethal concentrations 50 and 90 (LC_50_ and LC_90_) of the EO. The bioassay was conducted via an immersion methodology, as described by Santos et al. (2017) [29], in 24-well plates. Niclosamide was used as the positive control, and distilled water and dimethyl sulfoxide (1% DMSO) were used as the negative controls. Lethality was monitored after 24 and 48 h, and shell retraction and hemolymph release were observed to assess mortality. The experiments were performed in triplicate on at least three different days.

### 4.8. Algae Collection

Algae collection was carried out in the Jacaré River, Rio de Janeiro, RJ, Brazil (22°52′54.3′′ S 43°14′29.4′′ W), near the Oswaldo Cruz Foundation Campus. Three samples of approximately 200 mL were collected from the water column of the Jacaré River in sterilized vials and immediately transported to the Laboratory of Applied Studies in Photosynthesis, Institute of Chemistry, Federal University of Rio de Janeiro, Rio de Janeiro, RJ, Brazil. The samples were centrifuged, and the diversity of the local microflora in the precipitate was evaluated. Green microalgae, diatoms, and cyanobacteria were identified via optical microscopy. The microalgae strains were identified via optical microscopy on the basis of cell morphology. The precipitates containing the microalgae were resuspended in WC culture media [41]. To stimulate the growth of this biomass, it was transferred to fresh medium every seven days for three weeks. The medium was cultivated under a 12/12 h light and dark photoperiod at a temperature of 30 ± 2.0 °C with lateral illumination (150 µmol photons/m^2^/s on the outer surface of the vessels) provided by fluorescent lamps (Philips 23 W, white light).

#### 4.8.1. Isolation of Species (I) and Individual Cells (II)

The species were isolated by modifying the WC medium with other reagents, making it a selective medium. The selective medium for cyanobacteria was made by supplementing the original medium with 20 mg/mL cycloheximide. Cycloheximide is an antibiotic that inhibits the growth of most eukaryotes by interfering with 80S ribosomes during protein synthesis. Studies using cultures have confirmed the antibiotic activity of CHX against several eukaryotic algae and fungi and that CHX has little or no effect on prokaryotes such as cyanobacteria [42]. The selective medium for green algae (chlorophytes) was also prepared by adding 20 mg/mL penicillin G and 10 mg/mL germanium dioxide (GeO2) as additives to the original medium. Germanium dioxide (GeO2) has been commonly used in unialgal cultures of various seaweeds as an agent to eliminate diatoms. Researchers have reported that GeO2 prevents cell division in diatoms, usually by altering cell wall formation [43,44]. Penicillin G, on the other hand, prevents the growth of cyanobacteria by affecting cell wall formation. Single cells were isolated by serial dilution, consisting of 10 Falcon tubes containing 9 mL of culture medium, in which 1 mL of cell culture mixture was added to the first tube, which, after homogenization, was transferred to the next tube, and so on. Finally, tube 10 had a lower concentration of cells, resulting in a higher probability of isolating a single species. Using this method, we were able to isolate *Chlorella* spp., a green alga, and *Synechococcus* spp., a cyanobacterium. The microalgae were identified via light microscopy via a Zeiss microscope (Primostar3, Oberkochen, Germany).

#### 4.8.2. Cultivation of Isolated Species

The isolated species were inoculated into specific culture media: BBM [45] for *Chlorella* spp. and ASM-1 [46] for *Synechococcus* spp. All components of the culture media and glassware were autoclaved through a sterilization process involving the application of heat and pressure so that these objects were completely free of bacteria and microorganisms. The native microalgal strains were batch-cultured photoautotrophically in 2 L Erlenmeyer flasks containing 1.7 L of autoclaved medium. Cultures were exposed to lateral illumination (150 µmol photons m^−2^ s^−1^ on the outer surface of the vessels) provided by fluorescent lamps (Philips 23 W, white light) with a 12:12 h photoperiod. The culture flasks were maintained on a rotary shaker adjusted at 156 rpm (Lab-Line Instruments Inc., Odessa, TX, USA) at 30 ± 2 °C. The initial cellular density was 5.0 × 10^4^ cells. mL^−1^ for *Chlorella* spp. and *Synechococcus* spp. The cells were cultured under these conditions for 14 days before being subjected to the compounds tested in this study.

#### 4.8.3. Ecotoxicity Assay on Algae

The tests were performed in triplicate for each compound tested, with the substance added in different proportions to the algae (1:1, 1:2, and 1:4). The concentrations of the compounds used were based on the IC50 values already found in the tests on mollusks. After the addition of the substances, 10 mL of algae culture grown for 14 days was added. An aliquot of 70 μL was removed daily from each tube containing the algae culture supplemented with the compounds, and the cell viability was evaluated via optical microscopy, where the percentage of live cells was counted manually within the microscopic field. The response obtained was evaluated in terms of live cells or dead cells. The cells considered dead were those whose cell wall ruptured and whose intracellular contents had leaked into the external medium. All the views were compared with those of the control culture, which did not receive any compound. The cell viability responses were monitored for 7 days.

### 4.9. Ovicidal Assay

The bioassays were conducted via egg capsules collected from Styrofoam and subsequently placed in a *Biomphalaria glabrata* rearing tank located in the Lauro Travassos Pavilion at the Oswaldo Cruz Institute (IOC)/Fiocruz in Rio de Janeiro. The Styrofoam plates were collected from the rearing tank after 48 h. The egg capsules were carefully removed from the Styrofoam and transferred to a 24-well plate, following the methodology adapted from Santos et al. (2017) [29]. At the beginning of the experiment, or “time zero”, the viable eggs from each capsule were counted and distributed in each well containing the product to be tested. Niclosamide was used as a positive control, whereas distilled water and dimethyl sulfoxide (1% DMSO) were used as negative controls. The capsules were examined with a stereomicroscope (Fisher) 24 and 48 h after the beginning of the experiment. Disintegrated forms inside the eggs indicate embryo lethality. The experiments were performed in triplicate on at least three different days.

### 4.10. Cercaricidal Assay

A cercarial suspension of *Schistosoma mansoni* strain BH was obtained from *Biomphalaria glabrata* mollusks from the Malacology Reference Laboratory at the Oswaldo Cruz Institute (IOC)/Fiocruz. For preparation, 1000 µL of the cercarial suspension was pipetted into each well of a 24-well plate, and 40 µL of Lugol’s iodine was added. This dye stains the genetic material of the cercariae and kills them, allowing an approximate count of the cercariae per well via a stereomicroscope (Fisher).

For the cercaricidal activity experiment, 1 mL of EO from *Cymbopogon winterianus* leaves at concentrations of 50, 100, 150, and 200 mg/L was added to each well of the plate. Then, 1 mL of the cercariae suspension was added to each well. After that, 40 µL of Trypan blue dye was added. Readings were performed every hour for 4 h. Niclosamide was used as a positive control, and distilled water and dimethyl sulfoxide (1% DMSO) were used as negative controls. This experiment was repeated in triplicate and on at least three different days.

### 4.11. In Vivo Acute Toxicity Assay

Female Swiss mice were divided into ten experimental groups and six control groups to evaluate acute toxicity. The experimental group received the EO of *Cymbopogon winterianus* at single doses of 100 mg/kg and 1000 mg/kg orally and intraperitoneally. The control groups received saline or dimethyl sulfoxide (DMSO). After administration, the animals were monitored for 24 h to observe possible signs of toxicity, such as excessive agitation, ruffled fur, or mortality. The animals were euthanized with 50 μL of xylazine and 50 μL of ketamine intramuscularly 24 h after the last administration. All procedures were approved by the Ethics Committee on the Use of Animals from the Oswaldo Cruz Institute with registration number L043-2028-A1 on 21 December 2020.

#### Hematological and Biochemical Analysis

Cardiac puncture is a common technique for collecting blood from small rodents, such as mice. It allows rapid and efficient access to blood for hematological and biochemical analyses that evaluate the effects of the administered compounds.

After the 24 h acute toxicity test, the animals were continuously monitored and divided into three distinct groups. Group 1 received the control treatment with 0.9% saline solution (100 μL per animal). Group 2 was treated with control dimethyl sulfoxide (DMSO) (100 and 1000 mg/kg), and Group 3 received *Cymbopogon winterianus* EO (100 and 1000 mg/kg), which was administered intraperitoneally. After the observation period, euthanasia was performed with a solution of ketamine (300 mg/kg) and xylazine (30 mg/kg) intraperitoneally.

The animals were then placed in a supine position, and the thorax was cleaned and disinfected with 70% alcohol. A sterile syringe with a fine needle (0.45 × 13 mm) was used to perform a cardiac puncture, penetrating the thoracic cavity and puncturing the left atrium or ventricle of the heart. Blood was gently aspirated to avoid damage to the heart, with a maximum amount of 1 mL being collected for subsequent analyses.

The collected blood was divided into two samples: one for hematological analysis and the other for biochemical analysis. Approximately 300 μL was transferred to a tube with citrate as an anticoagulant for hematological analysis, while 500 μL was transferred to a 1.5 mL tube without anticoagulant for biochemical analysis.

Hematological and biochemical analyses were performed via automated colorimetric, kinetic, and potentiometric methodologies at the Institute of Science and Technology in Biomodels (ICTB), which is linked to the Oswaldo Cruz Foundation (Fiocruz).

### 4.12. Effect of Cymbopogon winterianus EO on Acetylcholinesterase

The activities of the enzymes acetylcholinesterase from *E. electricus* (eeAChE) and human AChE (hAChE), as well as human butyrylcholinesterase (hBChE), were evaluated via the Ellman method. The principle of this method consists of evaluating the rate of thiocholine production, as acetylthiocholine (ACTI) or butyrylthiocholine (BUTI) is hydrolyzed by AChE or BChE, respectively. Thiocholine reacts with 5,5′-dithiobis(2-nitrobenzoic) acid (DTNB), producing 5-thio-2-nitrobenzoic acid (TNB), a yellow anion, which can be quantified via a spectrophotometer at 412 nm [47]. Three independent assays were performed in triplicate, with a final volume of 250 μL in 96-well microplates containing phosphate buffer (0.05 M, pH 7 and 8), 10 mU/mL of the enzyme evaluated, 60 μM DTNB and samples (3 to 100 μg/mL), their respective vehicle DMSO (0.1 to 1%) or water (3 to 10%), and rivastigmine as a standard (50 μM). After 30 min under agitation protected from light, the reaction was initiated with the addition of ACTI or BUTI (1.5 mM), and the absorbance was measured at 412 nm for 10 min. The percentage of inhibition was determined by comparing the enzymatic reaction rates of the samples in relation to those of the control group.

### 4.13. Data Analysis

The results of the molluskicidal activity experiments were evaluated using means and standard errors in Excel via dispersion analysis to calculate the lethal concentrations. The ovicidal and cercaricidal activities were evaluated using means and standard errors and were analyzed via one-way ANOVA, with a significance level of *p* < 0.0001 compared with the negative control.

## 5. Conclusions

*Cymbopogon winterianus* EO demonstrated high efficacy as a molluskicide, successfully controlling the young mollusk *Biomphalaria glabrata* at a lethal concentration of 74.21 mg/L, as recommended by the WHO. In the histological analysis, no changes were observed in the tissues of mollusks treated with lethal concentration 25 (LC_25_). However, at lethal concentrations 50 (LC_50_) and 75 (LC_75_), crystalline concretions were detected near the renal saccular portion.

In addition, the EO was 90% lethal to *Schistosoma mansoni* cercariae after 4 h of exposure. Acute toxicity tests in mice revealed no signs of toxicity, and hematological and biochemical parameters remained within reference values, similar to those of the negative control. These results suggest that this EO is safe for use at the concentrations required for the control of mollusks and parasites.

The EO did not inhibit acetylcholinesterase (AChE), indicating low toxicity to fish, and caused a slight reduction in human butyrylcholinesterase (hBChE) activity without affecting human AChE, reinforcing its low toxicity to mammalian tissues. Although the oil did not have toxic effects on algae until the 75th day of observation, mortality began to be observed after this period, highlighting the importance of evaluating long-term effects, since algae are key bioindicators of water quality and ecological balance.

These results pave the way for future research, especially regarding the role of the monoterpenes citronellal, citronellol, and geraniol in molluskicidal activity. Understanding these mechanisms will be crucial for optimizing the application of EOs in mollusk control and interrupting the transmission cycle of schistosomiasis.

## Figures and Tables

**Table 1:** Chemical characterization of the EO of *Cymbopogon winterianus* via GC–MS and GC-FID.

Retention Time	Arithmetic Index	Arithmetic Index Calculated	Substances	%
7.029	974	991	β-pinene	0.1
8.264	1024	1028	Limonene	0.5
10.825	1095	1101	Linalool	0.6
12.630	1145	1146	Isopulegol	0.7
12.883	1148	1152	Citronellal	29.2
16.091	1223	1230	Citronellol	10.5
17.204	1249	1256	Geraniol	25.0
21.313	1350	1352	Citronellyl acetate	2.1
22.615	1379	1383	Geranyl acetate	3.7
22.930	1389	1391	β-elemene	1.6
26.557	1480	1479	Germacrene D	1.6
28.258	1528	1520	Zonarene	1.8
29.281	1548	1549	Elemol	9.6
30.288	1574	1575	Germacrene D-4-ol	1.2
32.820	1644	1642	Muurolol (=Torreyol)	1.4
33.298	1652	1655	α-Cadinol	3.3
**Total Identified**				92.9

**Table 2:** Lethal concentrations 50 and 90 of *Cymbopogon winterianus* EO against adult (10–12 mm) and young (6–8 mm/3–5 mm) *Biomphalaria glabrata* mollusks.

Stage of the Mollusk *Biomphalaria glabrata*	LC_50_ (mg/L)	LC_90_ (mg/L)
Adult (10–12 mm)	82.65	115.35
Young (6–8 mm)	60.62	74.21
Young (3–5 mm)	47.20	60.72

**Table 3:** Ecotoxicity assessment of *Cymbopogon winterianus* EO in *Chlorella* spp. (collectiona).

Substances	24 h	72 h	168 h	30 Days
	Survival (%)			
Control	100.0	100.0	100.0	100.0
*Cymbopogon winterianus* EO	100.0	100.0	100.0	100.0

**Table 4:** Ecotoxicity assessment of *Cymbopogon winterianus* EO in *Chlorella* spp. (isolated from mollusk hydrolysate).

Substances	24 h	72 h	168 h	75 Days
	Survival (%)			
Control	100.0	100.0	100.0	100.0
*Cymbopogon winterianus* EO	100.0	100.0	100.0	0.0

**Table 5:** Effects of acute oral administration and acute intraperitoneal administration of *Cymbopogon winterianus* EO in mice during 24 h of observation.

Acute Toxicity (24 Hours)				
Substance Used	Number of Mice	Sex	Mortality	Signs and Symptoms of Toxicity
Saline	10	Females	0/10	No animal showed signs of toxicity
Dimethyl sulfoxide (DMSO) 1000 mg/kg	15	Females	0/15	No animal showed signs of toxicity
Dimethyl sulfoxide (DMSO) 100 mg/kg	15	Females	0/15	No animal showed signs of toxicity
EO 1000 mg/kg	15	Females	0/15	No animal showed signs of toxicity
EO 100 mg/kg	15	Females	0/15	No animal showed signs of toxicity

**Table 6:** Hematological and biochemical analysis of Swiss Webster mice after treatment with 0.9% saline solution (100 μL/animal), dimethyl sulfoxide (DMSO), or *Cymbopogon winterianus* EO.

	Administrations			
	Saline Solution 0.9%	DMSO	EO	
Red Series				Reference Values
Red blood cell count	9.46	8.22	8.77	8.2 to 10.2 million/mm^3^
Hemoglobin dosage	14.4	13.4	13.9	14.4 to 15.7 g/dL
Hematocrit assessment	49.3	45.0	47.4	59.42 to 49.18%
Mean corpuscular volume	54.2	54.7	54.0	41.3 to 55.5 fm^3^
Mean corpuscular hemoglobin	15.2	16.3	15.8	14.9 to 16.5 pg
Average hemoglobin concentration	28.0	29.8	29.3	27.4 to 32.0 g/dL
**White series**				
Leukocyte count	4.1	4.2	4.9	3.8 to 5.7 thousand/mm^3^
**Platelet**				
Platelet Count	1038	1078	1122	901 to 1167 thousand/mm^3^
**Biochemistry**				
Albumin	2.6	2.4	2.8	2.5–4.8 g/dL
Calcium	9.0	8.5	8.5	5.9–9.4 mg/dL
Aspartate aminotransferase (AST)	467.0	73.0	700.0	59–247 U/L
Alanine aminotransferase (ALT)	320.0	31.0	515.0	28–132 U/L
Lactate dehydrogenase (LDH)	1234.0	460.0	1526.0	1105–3993 U/L
Alkaline phosphatase	156.0	204.0	185.0	62–209 U/L
Creatinine	<0.2	<0.2	<0.2	0.2–0.8 mg/dL

**Table 7:** Enzymatic activity of *Cymbopogon winterianus* EO.

	Enzymatic Activity (% Control)		
	eeAChE	hAChE	hBChE
	Mean ± Sepm	Mean ± Sepm	Mean ± Sepm
H_2_O	100.7 ± 2.5	100.7 ± 1.8	101.6 ± 0.9
DMSO (0.1%)	92.9 ± 2.3	99 ± 2.7	93.6 ± 3.7
Rivastigmine (50 µM)	1.4 ± 2.8	1.1 ± 0.4	7.2 ± 7.8
*C. winterianus* EO (100 µg/mL)	93.2 ± 3.9	103 ± 5.3	83.4 ± 4.4

## Data Availability

Data available on request from the authors.
